# A prospective, observational, cost of implementation analysis for the National Infection Surveillance Program for Aged Care (NISPAC)

**DOI:** 10.1186/s13756-026-01722-x

**Published:** 2026-06-05

**Authors:** Natasha Brusco, Sara Whittaker, Karin Thursky, David Dunt, Janet K. Sluggett, Ann Bull, Leon J. Worth, Noleen Bennett

**Affiliations:** 1 Rehabilitation, Ageing and Independent Living (RAIL) Research Centre, Monash University, Melbourne, Victoria Australia; 2 National Centre for Antimicrobial Stewardship, Department of Infectious Diseases, The University of Melbourne, Melbourne, Victoria Australia; 3 The Royal Melbourne Hospital Guidance Group, Melbourne Health, Melbourne, Victoria Australia; 4 Sir Peter MacCallum Department of Oncology, The University of Melbourne, Parkville, Victoria Australia; 5 Professor Emeritus, The University of Melbourne, Parkville, Victoria Australia; 6 Adelaide University, School of Allied Health and Human Performance, College of Health, Adelaide, South Australia Australia; 7 South Australian Health and Medical Research Institute, Adelaide, South Australia Australia; 8 Victorian Healthcare Associated Infection Surveillance System (VICNISS) Coordinating Centre, Melbourne, Australia; 9 Department of Medicine, The University of Melbourne, Parkville, Victoria Australia; 10 Department of Nursing, Melbourne School of Health Sciences, The University of Melbourne, Parkville, Victoria Australia

## Abstract

**Background:**

The National Infection Surveillance Program for Aged Care (NISPAC) is a new standardised and streamlined infection and antimicrobial use surveillance tool for Australian Residential Aged Care Homes (RACHs). As a part of the program development and implementation, it was identified that the cost of NISPAC implementation in Australian RACHs was currently unknown, and the objective of this study was to determine this cost from the perspective of the Australian Aged Care setting.

**Methods:**

The cost of implementation included those of the three coordinating bodies, as well as the individual RACHs who participated in the NISPAC program implementation. Development and implementation resources and costs for the coordinating bodies were determined over a 3 year period and then attributed equally across the participating RACHs. Participating RACHs (*n* = 69) reported the resources and cost of NISPAC implementation over a 5 month period, and this included salary and non-salary costs (2 months pre-implementation and 3 months of implementation). Costs for the participating RACHs were added to the costs of the coordinating bodies to determine the total cost per RACH. The price year for the analysis was 2024.

**Results:**

The total implementation cost for the three coordinating bodies was $AUD1,869,279, resulting in a mean cost attributed per RACH of $AUD27,091 (SD $AUD0). The mean cost of RACH staff time for the intervention during the pre-implementation planning phase was $276.18 (SD $AUD219.55), and during the implementation phase was $AUD1,146.47 ($AUD1,003.48). The mean cost of NISPAC implementation per RACH was $AUD28,805 (SD $AUD1,137).

**Conclusion:**

The novel NISPAC was a relatively low-cost infection and antimicrobial use surveillance program to implement in RACHs. Future studies are required to establish the cost-effectiveness of the program.

Keywords: Infection, surveillance and cost analyses

## Background

Since 2020, a group of leading Australian health services researchers have collaborated to develop and implement the novel National Infection Surveillance Program for Aged Care (NISPAC) [1–4]. The NISPAC program was developed via consumer and subject expert consultation, in response to the high levels of infection experienced by older people in Australian residential aged care homes (RACHs) [4]. Many infections experienced in RACHs are potentially preventable through dedicated programs focussed on optimising infection prevention and control (IPC) practices [1, 2] It was intended that NISPAC will streamline and standardise infection and antimicrobial use surveillance methodology, and for the first time in Australia, enable program evaluation and national benchmarking [1].

Despite the benefit of infection and antimicrobial use surveillance in RACHs as an IPC strategy, barriers exist to uptake [3]. Prior to NISPAC, there were two other infection and antimicrobial use surveillance programs available in Australia (Antimicrobial Use and Resistance in Australia (AURA) and National Antimicrobial Utilisation Surveillance Programs (NAUSP), however, these programs are not mandated or available to all RACHs [3]. Focus groups involving RACH staff that were undertaken to evaluate the two existing surveillance programs revealed several key barriers to uptake, including the time required to collect and enter the data, competing priority tasks and limited staff understanding of surveillance. Another barrier is the unknown cost to the RACH of implementing a comprehensive infection and antimicrobial use surveillance program such as NISPAC, which aims to reduce infections and improve antimicrobial use within aged care services by monitoring and reporting on infections and antimicrobial usage (10 modules, across 5 categories), promoting best practices through education and support for all staff involved in infection prevention, control, and antimicrobial stewardship, and enhancing the quality of care in RACHs through standardized, data-driven program delivery [1]. Participating RACHs could select one or more of the five categories (10 modules), noting all surveillance modules chosen needed to be completed within that category. For the significant organism and resident vaccination categories there were multiple modules requiring completion.

There is increasing focus on program and economic evaluations for infection prevention, control, and antimicrobial stewardship programs in RACHs, particularly national programs which are subsidised [5]. An economic evaluation examines the costs and consequences of alternate interventions [6]. Consequences include the impact of the intervention, and in the context of NISPAC this may comprise the number and severity of infections. Costs may include the intervention development costs, intervention implementation costs, and intervention treatment costs [7] Standalone reporting of the intervention implementation costs, via a cost of implementation study (a cost analysis), is relatively new, as prior to the year 2000 cost of implementation studies were scarce and most often of poor quality [7]. Over the past two decades cost of implementation studies have been more regularly reported in peer-reviewed journals, with several methodological papers available to guide the development of a cost of implementation study, noting that a cost of implementation study should still adhere to the overarching Consolidated Health Economic Evaluation Reporting Standards (CHEERS 2022) [6, 8, 9].

NISPAC is currently aiming to develop, implement and evaluate an improved, standardised and streamlined infection and antimicrobial use surveillance tool for Australian RACHs. As a part of the NISPAC program development and implementation, it was identified that the cost of NISPAC implementation was of interest [5] yet currently unknown, and as such, the aim of this evaluation was to establish the cost of implementing and sustaining NISPAC per RACH. This study gives insight and understanding into implementation costs across the representative sample of RACHs who participated in the implementation of NISPAC, which will help inform future national scaling of the intervention.

## Methods

This cost of implementation study has been reported according to CHEERS [6] (Appendix 1) and it received ethical approval from the Royal Melbourne Hospital Human Research Ethics Committee (HREC) (HREC/82249/MH-2022).

Through a standardised, data-driven approach, NISPAC aims to improve the quality of care in RACHs. Central to its focus is the reduction of infection rates and the optimisation of antimicrobial use. This is achieved by collecting and analysing data across 10 modules within five key categories, providing targeted education and support to staff involved in infection prevention, control, and antimicrobial stewardship, and fostering the consistent implementation of best practices across aged care services [1]. Participating RACHs use a mixture of online and paper based data collection, and are located in both metropolitan and rural areas.

The cost of implementation included those incurred by the individual RACHs and the three NISPAC coordinating bodies (i.e., VICNISS, Victorian Healthcare Associated Infection Surveillance System; NCAS, National Centre for Antimicrobial Stewardship; and ROSA, the Registry of Senior Australians).

### Study population

The study population were the RACH staff (mostly nurses) who were involved in the implementation of NISPAC. The study population also included VICNISS, NCAS and ROSA employees who were involved in the development or implementation of NISPAC.

### Setting and location

The setting was 69 RACHs located in metropolitan, regional and rural areas of Australia.

### Comparators, outcomes and modelling

This cost analysis did not include comparators, outcomes or economic modelling.

### Perspective, time horizon, discount rate currency, price date and conversion

In addition to the 3 year preparation costs from the coordinating bodies (VICNISS, NCAS and ROSA), this cost of implementation study adopted the perspective of the Australian Aged Care system (including the stakeholders of the Australian government in the aged care setting) over a 5 month time horizon: (1) Pre-implementation planning phase where staff underwent training and systems were put in place (June and July 2024), and (2) Implementation phase where active surveillance commenced and data was collected (August–October 2024), noting that over this three 3 month period the RACHs mostly did paper based NISPAC data collection. Discounting was not applied due to the short time horizon. The price date was the $AUD 2024 calendar year with all costs prior to this inflated by CPI [10].

### Measurement and valuation of resources and costs

Appendix 2 and 3 are the data collection tools for the participating RACHs and these provide details for each of the cost categories. Data collection tools (Appendix 2 and 3) were distributed via email by the research team to key nominees at each RACH, these were subsequently returned by email by each participating facility. Appendix 2 is the data collection form for the pre-implementation (i.e. preparation) period and Appendix 3 is the data collection form for the implementation period. Preparation costs included: (i) training costs (e.g. attendance at LAUNCH session, familiarisation with NISPAC ‘user folder’, familiarisation with NISPAC IT platform, and/or observation of NISPAC training videos); (ii) planning costs (e.g. completion of NISPAC annual surveillance plan, and/or creation of summary database for vaccination records); (iii) costs of development and access of data source systems (e.g. development of (potential) infection or ‘antimicrobial starts’ notification system, accessibility to laboratory summary reports, and/or organisation of information technology support); (iv) communication costs (e.g. staff costs to communication with residents and carers about the pilot NISPAC, communication with others involved in data collection/submission, communication with managers and relevant committees about the pilot NISPAC, and/or communication with NISPAC team) ; (v) other (i.e. not otherwise specified). Staff time from RACHs was valued based on the Nurses and Midwives 2020–2024 Enterprise Bargaining Agreement (EBA) 11, with Aged Care Registered Nurse (Level 3) classification selected for the value of staff time ($48.12 per hour), with an additional 25% organisational oncosts applied to the base rate ($48.21 per hour plus 25% = $60.15 per hour).

Staff time from VICNISS, NCAS and ROSA was valued based study budget, inflated to the reporting year of the evaluation. Market rates were applied to all other non-salary costs based on the study budget.

### Analytics and assumptions

Costs for the participating RACHs were added to the costs of the coordinating body to determine the total cost per RACH. The costs incurred by the RACH is considered a variable cost depending on the number of modules implemented, whereas the costs of the co-ordinating body resources were considered a fixed cost and were spread evenly across the participating RACH. Resources and costs are presented as a mean and standard deviation.

Assumptions are detailed in Appendix 4. Participating RACHs reported the resources and cost of NISPAC implementation, including salary and non-salary costs. Development and implementation resources and costs for the coordinating bodies (VICNISS, NCAS and ROSA) were determined by the NISPAC project budget, and supplemented by interviews to explore costs for each organisation, and then attributed equally across the participating RACHs.

### Heterogeneity, distributional effects and uncertainty

A post-hoc exploratory analysis was completed to extrapolate costs for sustained implementation of NISPAC over a 12 month period. Costs were extrapolated to 12 months to provide insights for scaling, noting that often financial budgeting for RACH are across a 12 month period. Economic assumptions were adopted. These included modelling 3 year co-ordinating body costs to remove costs that were not expected to be required during ongoing rollout were excluded with the remainder 3 year costs divided by three to represent 12 months; the RACH 3 month implementation costs were multiplied by four to reflect the expected costs to the RACH over a 12 month period (Appendix 4). Costs were presented as a mean and standard deviation.

### Engagement with others affected by the study

The cost of implementation survey was tested by three staff from a participating RACH, prior to roll-out for data collection. Feedback from these staff led to simplification of the survey to reduce the detail collected by each RACH.

## Results

All 69 RACHs that officially participated in the implementation of NISPAC were included in the cost of implementation analysis. The number of beds in each RACH ranged from 7 to 285 (mean 76.88, SD 54.53). Of the 69 RACHs, 41 were metropolitan (59%) and 28 were rural or regional (41%); and they were located in Victoria (*n* = 29, 24%), New South Wales (*n* = 6, 9%), Western Australia (*n* = 3, 4%), Queensland (*n* = 15, 22%), Tasmania (*n* = 1, 2%), South Australia (*n* = 12, 17%), and Australian Capital Territory (*n* = 3, 4%). In addition, 27 homes (39%) participated in the Significant Organism Module, 37 homes (54%) participated in the staff vaccination module, 40 homes (58%) participated in the resident vaccination module, 31 homes (46%) participated in the urinary tract infection module, and 43 homes (64%) participated in the Aged Care National Antimicrobial Prescribing Survey (ACNAPS), noting that an additional 15 homes (22%) did complete this module also, however, it was outside of the implementation period of the study, and not included in the evaluation. There were five homes (7%) that did not complete any of the modules during the evaluation period.

There were 31 homes (45%) who did not submit a pre-implementation cost survey, and one of the homes provided only 1 month of data requiring imputation of data as per economic assumptions (Appendix 4). For the implementation period, 49 homes (71%) did not submit a cost of implementation survey; of those that did (*n* = 20), eight required imputation of data as per economic assumptions for one of the months of data (Appendix 4). For those homes that had missing data, their characteristics have been described in Appendix 5.

The mean cost of RACH staff time for the intervention during the 2 month pre-implementation planning phase was $276.18 (SD $219.55), and the mean cost to the RACH during the 3 month implementation active phase was $1146.47 ($1003.48) (Table 1). Implementation costs were compared between metropolitan and rural/regional RACHs, noting implementation costs were less at rural/regional RACHs (Table 1). The total cost to implement the intervention to the co-ordinating body funders (VICNISS, NCAS and ROSA) was $1,869,279, and the mean per RACH was $27,091 (SD $0) with the costs to the funders being equally distributed across all RACH (Table 2). The total cost of NISPAC implementation considers both the RACH resources, and the co-ordinating body resources, and this was $28,805 (SD $1137) (Table 3). Comparable costs for implementing NISPAC at RACH in metropolitan versus regional/rural areas were observed (Table 3).

A post-hoc exploratory analysis was conducted to extrapolate the cost per year for continued implementation of the program, modelling the RACH implementation costs and co-ordinating body costs to be reflective of a 12 month period (Table 4). The mean cost per RACH considering both cost contributions from the RACH (mean $4585 (SD $2122), and co-ordinating body costs (mean $5177 (SD $0)), was $10,362 ($2122) per year. A post-hoc sensitivity analysis was also completed, including only RACH (*n* = 14) with completed data, the mean cost per year for continued implementation was estimated to be $11,530 (SD $4260).

**Table 1:** RACH resources for implementation

	Resources: staff time (minutes), mean (SD)			Cost of staff time (AUD), mean (SD)		
	Metropolitan	Regional/rural	Combined	Metropolitan	Regional/rural	Combined
Pre-implementation planning phase, n	21	17	38	21	17	38
Training	103 (87)	151 (68)	125 (82)	$102.92 ($87.48)	$151.40 ($68.10)	$124.61 ($82.09)
Planning	31 (39)	48 (40)	39 (40)	$31.37 ($39.36)	$47.72 ($39.60)	$38.68 ($39.80)
Data source systems	29 (75)	33 (34)	31 (59)	$29.05 ($74.74)	$32.94 ($33.76)	$30.79 ($59.30)
Communication	70 (67)	97 (132)	82 (101)	$70.00 ($66.96)	$97.06 ($131.71)	$82.11 ($100.55)
Other	0 (0)	0 (0)	0 (0)	$0.00 ($0.00)	$0.00 ($0.00)	$0.00 ($0.00)
Total	233 (210)	329 (226)	276 (220)	$233.33 ($209.95)	$329.12 ($225.79)	$276.18 ($219.55)
Implementation phase, n	10	10	20	10	10	20
Data collection and submission	830 (623)	482 (383)	656 (534)	$830.32 ($623.17)	$481.74 ($383.38)	$656.03 ($534.37)
Training	104 (168)	114 (181)	109 (170)	$103.54 ($168.08)	$113.79 ($180.50)	$108.67 ($169.83)
Communications about pilot NISPAC	388 (897)	355 (593)	371 (740)	$387.60 ($896.60)	$354.72 ($592.60)	$371.16 ($739.88)
Other	5 (13)	16 (30)	11 (23)	$5.11 ($13.03)	$16.11 ($29.99)	$10.61 ($23.20)
Total	1327 (863)	966 (1,144)	1,146 (1,003)	$1326.58 ($862.82)	$966.36 ($1,144.23)	$1146.47 ($1,003.48)

Costs reported in AUD 2024 calendar year

**Table 2:** Co-ordinating body (VICNISS, NCAS and ROSA) resources and costs for initial implementation

	Resources (EFT)		Costs	
	Total (sum)	Per RACH *n* = 69 (mean (SD))	Total (sum)	Per RACH *n* = 69 (mean (SD))
VICNISS (combined)	–	–	$1,523,865	$22,085 ($0)
Project manager	0.8 FTE	0.01 (0.0)	$382,260	$5540 ($0)
IT support	1.0 FTE	0.01 (0.0)	$195,339	$2831 ($0)
Research fellow	1.0 FTE	0.01 (0.0)	$327,819	$4751 ($0)
Pharmacist	0.4 FTE	0.01 (0.0)	$165,600	$2400 ($0)
Personnel support	–	–	$89,907	$1303 ($0)
Equipment	–	–	$75,417	$1093 ($0)
Consumables^†^	–	–	$4623	$67 ($0)
Partner funding	–	–	$276,828	$4012 ($0)
Stage 1 Interviews: Staff time	95 min	1 min	$95	$1 ($0)
Other	–	–	$6003	$87 ($0)
NCAS (combined)	–	–	$222,939	$3231 ($0)
Personnel support	–	–	$89,907	$1303 ($0)
Consumables^†^	–	–	$4623	$67 ($0)
Partner funding	–	–	$122,337	$1773 ($0)
Stage 1 interviews: staff time	73 min	1 min	$75	$1 ($0)
Other	–	–	$6003	$87 ($0)
ROSA (combined)^¶^	–	–	$122,475	$1775 ($0)
Project officer	–	–	$63,342	$918 ($0)
Consumables^†^	–	–	$4623	$67 ($0)
Partner funding	–	–	$34,569	$501 ($0)
Other			$19,941	$289 ($0)
Total	–	–	$1,869,279	$27,091 ($0)

^¶^Costs included under the co-ordinating body ROSA, do not include the in-kind contributed including data linkage, the working environment used for the analysis, and the personnel involved with maintaining the registry

^†^Consumables, include computers required for personnel

**Table 3:** Combined RACH and co-ordinating bodies (VICNISS, NCAS and ROSA) costs for implementation

	Total costs (sum)			Cost per facility (Mean (SD))		
	Metropolitan (*n* = 6)	Rural/regional (*n* = 8)	Combined (*n* = 14)	Metropolitan (*n* = 6)	Rural/regional (*n* = 8)	Combined (*n* = 14)
RACH	$14,927	$17,507	$32,434	$2,487 ($2,284)	$2,188 ($2,191)	$2,316 ($2,149)
VICNISS	$132,510	$176,680	$309,190	$22,085 ($0)	$22,085 ($0)	$22,085 ($0)
NCAS	$19,386	$25,848	$45,234	$3,231 ($0)	$3,231 ($0)	$3,231 ($0)
ROSA	$10,650	$14,200	$24,850	$1,775 ($0)	$1,775 ($0)	$1,775 ($0)
Total	$173,792	$229,472	$403,264	$28,965 ($885)	$28,684 ($1,343)	$28,805 ($1,137)

Only including RACHs that provided RACH pre-implementation and implementation costs (n = 14)

**Table 4:** Extrapolated 12 month ongoing implementation costs

	Costs per RACH mean (SD)		
	Metropolitan (*n* = 41)	Regional/Rural (*n* = 28)	Combined (*n* = 69)
RACH	$4,760 ($1667)	$4327 ($2666)	$4585 ($2122)
Co-ordinating body	$5777 ($0)	$5777 ($0)	$5777 ($0)
Total	$10,537 ($1667)	$10,105 ($2666)	$10,362 ($2122)

## Discussion

Our main study finding was that NISPAC implementation resulted in a cost of $28,805 per RACH, with the majority of the cost being attributed to the set-up costs of the co-ordinating bodies. Hence the NISPAC program can be considered a relatively low-cost infection and antimicrobial use surveillance program. Cost to implement NISPAC in regional/rural areas was comparable to metropolitan areas. However, these implementation costs are conservative as it has not included in-kind contributions by the research team outside of the project budget, which provided ongoing governance to the project. In addition, this cost of implementing NISPAC was minimised by using data system infrastructure that was already established as part of the two existing surveillance programs. The broader project also leveraged linked national health and aged care data from the Registry of Senior Australians data platform, which has required considerable infrastructure grant funding and resources to establish and maintain [12], and it should be noted that these costs are not captured in this study (i.e. the economic costs associated with ROSA are conservative, not capturing this in-kind support).

Future scaling of NISPAC can be informed by the extrapolation completed as part of this evaluation, with the expected costs for ongoing implementation being $10,362 per year per RACH. The findings can be generalised to future programs. Based on 2617 RACHs currently operating in Australia, providing 223,691 residential beds [13], the annual cost to run NISPAC across all RACHs in Australia would approximate $27MIL.

Future planning for the NISPAC program may include streamlining the involvement of the three NISPAC co-ordinating bodies, now that NISPAC has been setup to manage ongoing rollout. This future direction has the potential to reduce the cost of ongoing implementation as the majority of the implementation cost was attributed to the set-up costs of the co-ordinating bodies. Another consideration for future planning is the use of electronic versus paper-based data collection for the surveillance data. While the current study allowed both, future scaling would at least rely primarily on electronic data submission due to its greater efficiencies. As part of the pilot study, data was collected on hard copy data collection forms. Data was submitted via webform for one module (resident influenza vaccination) only. Data for the other modules was emailed to and entered onto Excel spreadsheets by NISPAC staff. Advantages of using a webform included increased efficiency, improved data quality through automated data entry validation, and real time access to submitted data.

Strengths of this study includes data being collected from RACHs from 6 states across Australia, inclusive of both regional/rural and metropolitan RACHs, giving support to an informed estimation of national implementation costs. To the authors knowledge, this is the only antimicrobial surveillance cost of implementation study in RACH. Limitations to this study include using a representative sample of RACHs to estimate the cost of NISPAC implementation, only including 5 months of data collection, and having missing data that required imputation. Possible reasons for the non-completion of surveys during the implementation period included time constraints, competing priorities or work demands that may have meant the RACH were unable to continuously monitor the time taken to complete surveillance activities during busy periods of the project. Given this, there is also the potential of non-response bias in the results. Further research should include a cost-effectiveness analysis, as well as a break-even analysis, to understand the number of infections that would need to be avoided to break even with the cost of implementation.

## Conclusion

The novel NISPAC is a relatively low-cost infection and antimicrobial use surveillance program. Future fully powered studies are required to establish the cost-effectiveness of the program.

## Data Availability

Data is available on reasonable request to the corresponding author.

## Appendix 1: CHEERS checklist

**Table:**

Topic	No	Item	Location where item is reported
Title	1	Identify the study as an economic evaluation and specify the interventions being compared	Page 1
Abstract	2	Provide a structured summary that highlights context, key methods, results, and alternative analyses	Page 2
Introduction			Page 4–5
Background and objectives	3	Give the context for the study, the study question, and its practical relevance for decision making in policy or practice	Page 5–6
*Methods*			
Health economic analysis plan	4	Indicate whether a health economic analysis plan was developed and where available	Page 10
Study population	5	Describe characteristics of the study population (such as age range, demographics, socioeconomic, or clinical characteristics)	Pag3 7
Setting and location	6	Provide relevant contextual information that may influence findings	Page 8
Comparators	7	Describe the interventions or strategies being compared and why chosen	Page 8
Perspective	8	State the perspective(s) adopted by the study and why chosen	Page 8
Time horizon	9	State the time horizon for the study and why appropriate	Page 8
Discount rate	10	Report the discount rate(s) and reason chosen	Page 8
Selection of outcomes	11	Describe what outcomes were used as the measure(s) of benefit(s) and harm(s)	Page 8–9
Measurement of outcomes	12	Describe how outcomes used to capture benefit(s) and harm(s) were measured	Page 8–9
Valuation of outcomes	13	Describe the population and methods used to measure and value outcomes	Page 9
Measurement and valuation of resources and costs	14	Describe how costs were valued	Page 9
Currency, price date, and conversion	15	Report the dates of the estimated resource quantities and unit costs, plus the currency and year of conversion	Page 8
Rationale and description of model	16	If modelling is used, describe in detail and why used. Report if the model is publicly available and where it can be accessed	N/A
Analytics and assumptions	17	Describe any methods for analysing or statistically transforming data, any extrapolation methods, and approaches for validating any model used	Page 10
Characterising heterogeneity	18	Describe any methods used for estimating how the results of the study vary for subgroups	Page 10
Characterising distributional effects	19	Describe how impacts are distributed across different individuals or adjustments made to reflect priority populations	Page 10
Characterising uncertainty	20	Describe methods to characterise any sources of uncertainty in the analysis	Page 10
Approach to engagement with patients and others affected by the study	21	Describe any approaches to engage patients or service recipients, the general public, communities, or stakeholders (such as clinicians or payers) in the design of the study	Page 11
*Results*			
Study parameters	22	Report all analytic inputs (such as values, ranges, references) including uncertainty or distributional assumptions	Page 12
Summary of main results	23	Report the mean values for the main categories of costs and outcomes of interest and summarise them in the most appropriate overall measure	Page 13
Effect of uncertainty	24	Describe how uncertainty about analytic judgments, inputs, or projections affect findings. Report the effect of choice of discount rate and time horizon, if applicable	Page 12
Effect of engagement with patients and others affected by the study	25	Report on any difference patient/service recipient, general public, community, or stakeholder involvement made to the approach or findings of the study	Page 13
*Discussion*			
Study findings, limitations, generalisability, and current knowledge	26	Report key findings, limitations, ethical or equity considerations not captured, and how these could affect patients, policy, or practice	Page 18–19
*Other relevant information*			
Source of funding	27	Describe how the study was funded and any role of the funder in the identification, design, conduct, and reporting of the analysis	Page 20
Conflicts of interest	28	Report authors conflicts of interest according to journal or international committee of medical journal editors requirements	Page 20

## Appendix 2

Cost Implementation Study (1): June-July 2024.

NISPAC preparation months (pre-implementation).

*Instructions*: During July, you may use the spreadsheet on pages 2 and 3 to determine the ‘preparation cost’ for your RACH to participate in the pilot NISPAC. Please record the total number of minutes your team spent each week on items that relate to Training, Planning, Data source systems, Communication, and other NISPAC related activities. At the end of July, you will be requested to submit the total number to NISPAC for each item. For the Data source system section, please used the ‘Current status column’ to classify the status of each listed item as either (1) Done (completed in preparation for NISPAC), (2) Completed prior (Already in place in your RACH), (3) Not necessary (for the module(s) on our surveillance plan) or (4) Working on it.

An example of a completed form is outlined on the following page.

**Table:**

**Example only** tasks (minutes)		Current status	Jun 24th–30th	Jul 1st–7th	Jul 8th–14th	Jul 15th–21st	Jul 22nd–-28th	Jul 29th–31st	Total (mins)
Training	Attendance at LAUNCH session (on the day or recording)		60						60
	Familiarisation with NISPAC ‘User folder’			30					30
	Familiarisation with NISPAC IT platform			30					30
	Observation of NISPAC training videos		25		30				55
	Registration of RACHs (via NISPAC platform)			20					20
Plan	Completion of NISPAC annual surveillance plan			25					25
	Creation of vaccination records (summary database)								0
Data source systems	Development of (potential) infections notification system (line list)	Done	30						30
	Development of ‘antimicrobial starts’ notification system	Completed prior							0
	Accessibility to laboratory summary reports	Not necessary				25			25
	Organisation of information technology support	Working on it		10					10
Communication	With residents and carers about the pilot NISPAC				50				50
	With others who will be directly involved in data collection and/or submission					10			10
	With managers and relevant committees about the pilot NISPAC			10			10		20
	With the NISPAC team								0
*Other*									
Total	Not applicable	NA							**365**
*Did you spend money on equipment or other items to participate in the pilot NISPAC?’ Yes/No, If yes, please list the equipment and the value in AUD*									
Equipment description									Cost (AUD)
iPad									$850.00
Other									$0.00
Total									**$850.00**

## Appendix 3

Cost Implementation Study: Aug-Oct 2024.

NISPAC live months (implementation).

*Instructions*: During *August–October*, you may use the following worksheets to determine the cost for your residential aged care home (RACH) to participate in the pilot NISPAC. Please record the total number of minutes you or your team spent each week on items that relate to Data collection and submission, Training, Communication, and other NISPAC related activities.

An example of a completed worksheet is outlined on the following page.

**Table:**

**Example only** tasks (minutes)		Nov 1st–3rd	Nov 4th–10th	Nov 11th–17th	Nov 18th–24th		Nov 25th–30th	Total (mins)
Data collection and submission	Aged care national antimicrobial prescribing survey (NAPS)	30	75					105
	Resident vaccination modules (COVID-19, herpes zoster, influenza and pneumococcal disease)	Not participating						–
	Staff influenza vaccination module	Not participating						–
	Significant organism modules (MRSA infection, VRE infection and CDI)	10	15	10	35		10	80
	Urinary tract infection module		15		45		10	70
Training	Observation of NISPAC training videos		35					35
	*Other*							–
Communication about the pilot NISPAC	With residents and carers			10				10
	With others directly involved in data collection and/or submission		20					20
	With managers and relevant committees			10				10
	With the NISPAC team	10					10	20
Other								–
Total		50	160	30	80		30	**350**
*Did you spend money on equipment or other items to participate in the pilot NISPAC?’ Yes/No, If yes, please list the equipment and the value in AUD*								
Equipment description						Cost (AUD)		
Additional desk top computer at the nurses station						$1000.00		
Other						$0.00		
TOTAL						**$1000.00**		

## Appendix 4: Economic assumptions

**Table:**

Economic assumptions made	Detail of assumption
Cost year	2024 calendar year
Salary costs: Staff time from RACH (Infection prevention and control (IPC) Leads)	Staff time reported from participating RACHs included both enrolled nurses and registered nurses as IPC leads. It was reported by participating RACHs that of those involved in NISPAC implementation, two thirds (67%) were registered nurses, and one third (33%) enrolled nurses. Therefore, to extrapolate salary costs of nursing staff time to implement the intervention, registered nurses were used to model costs. Staff time from RACHs was valued based on the current Nursing Enterprise Bargaining Agreement (EBA) Enterprise agreements | Fair Work Commission with Aged Care Registered Nurse (Level 3) classification selected for the value of staff time ($48.12 per hour), with an additional 25% organisational oncosts applied to the base rate ($48.21 per hour plus 25% = $60.15 per hour). Therefore, the staff costs per minute was $1.00.
Non-salary costs	Market rates were applied to all other non-salary costs as appropriate.
Costs associated with development and implementation of resources	Development and implementation resources and costs for the coordinating bodies (NISPAC, VICNISS, NCAS and ROSA) will be determined for the NISPAC project, and then attributed equally across the participating RACHs.
Cost extrapolation included in the discussion: Number of RACH	The number of registered RACH in Australia was based on the most recent version of the Australian Aged Care service list https://www.gen-agedcaredata.gov.au/topics/providers,-services-and-places-in-aged-care 43
Research costs	Research costs supporting the implementation have been included in the cost analysis, as they were seen to be important part of the intervention implementation (i.e. hybrid design of effectiveness and implementation). Research costs for the evaluation have been excluded from the cost analysis.
Inclusion of data	For participating homes that submitted preparation data, and withdrew during the active phase, preparation data was included in the cost of implementation, however, they were not included in the cost of implementation for the active phase. Participating homes that did not submit demographic data survey were removed from the analysis. Participating wards must have reflected they were participating in one (or more of the surveillance modules) to be included in the analysis (i.e. for homes that did not complete any modules, there shouldn’t be any cost of implementation data collection tools submitted). If pre-implementation data was submitted, this was included in the evaluation. Some participating organisations submitted one data collection form including multiple homes within that organisation, when this occurred responses was divided across the homes evenly.
Data submitted outside of data collection periods	If an active phase of data was submitted outside of the study period being August to October (e.g. in November), the data submitted outside of was not included in the analysis.
Missing data	For the main dataset provided by researchers to health economists, where the participating homes nominated a duration of time for some of the data variables but left others blank, it is assumed that these blanks were ‘0’ minutes. This was imputed by health economists. If there is missing data for 1 month of the active phase, health economists imputed the missing data for that facility from the average of the submitted data. If the June preparation data was missing for a facility, it was assumed that the facility did not start preparation until July (*n* = 1 facility that this applied to). There was only 6 days of preparation time in June following the training on the 25/6/24. If there was only 1 month of data during the active, health economists have imputed the missing data for that facility from the average of the submitted data from all homes for that month.
In-kind contributions	In-kind contributions above and beyond what was anticipated as part of the project budget is noted to be a conservative estimate. Additional in-kind contributions over the budget have not been included in this analysis. For example, for the co-ordinating body ROSA, the costs of accessing the data, the working environment used for analysis, and the personnel involved with maintaining the registry were not included in this cost of implementation analysis, making the estimate for ROSA conservative.
Preparation data collected	Training for the intervention was on 25/6/2024, this is the assumed start date for the preparation phase of data collection. Therefore, there was only 6 days of data collection in June 2024, compared to the full month of July 2024.
Consumable costs captured as costs incurred by co-ordinating bodies	Consumable costs included computers.
Additional VICNISS server capacity and software licence	The costs for the additional VICNISS server capacity and software licence have been included under equipment in the cost of resources.
Other costs incurred by co-ordinating bodies	Other costs incurred by co-ordinating bodies include: SURE Data access, Ethics, Consumer payments, travel
Costs not allocated to a co-ordinating body on the budget	Costs not allocated to a co-ordinating body in accordance with the budget has been allocated as appropriate to co-ordinating bodies through an interview with the CIA.
Costs to homes to cover costs of co-ordinating bodies as part of implementation	Co-ordinating body costs have been equally spread over the 69 homes who committed to participate at the start of the study.
Missing cost of implementation data for RACH resources	For the RACH that did not submit data collection forms for the cost of implementation analysis, the mean costs for pre-implementation and active phases were imputed to extrapolate the cost of implementation across the 69 participating RACH. This was used in the extrapolation of costs for ongoing implementation for a 12 month period.
Fixed and variable costs	The total cost of implementation considers both the RACH resources, and the co-ordinating body resources. The costs incurred by the RACH is considered a variable cost depending on the number of modules implemented, whereas the costs of the co-ordinating body resources were considered a fixed cost and were spread across the 69 participating RACH.
Extrapolation of ongoing costs for 12 months	Economic assumptions were adopted. These included modelling 3 year co-ordinating body costs to remove costs that were not expected to be required during ongoing rollout were removed with the remainder 3 year costs divided by three to represent 12 months; the RACH 3 month post-implementation costs were multiplied by four to reflect the expected costs to the RACH over a 12 month period.
Rurality	Rurality was determined using aged care service list classification, accessible via: aged care services list 2024

## Appendix 5: RACH characteristics

**Table:**

	Characteristics of RACH with included cost of implementation data	Characteristics of RACH without cost of implementation data
Pre-implementation phase	*n* = 38	*n* = 31
*Provider type* (n, %)		
Not-for-profit	27 (71%)	15 (48%)
Public	10 (26%)	14 (45%)
Private	1 (3%)	2 (6%)
*Rurality* (n, %)		
Metropolitan	21 (55%)	20 (65%)
Regional/rural	17 (45%)	11 (35%)
*Bed number* (mean (SD))	87 (63.17)	66 (40.1)
*Module participation* (n, %)		
Significant organism	21 (57%)	6 (19%)
Staff vaccination	23 (62%)	14 (45%)
Resident vaccination	28 (76%)	12 (39%)
UTI	17 (46%)	14 (45%)
Active phase	*n* = 20	*n* = 49
*Provider type* (n, %)		
Not-for-profit	11 (55%)	31 (63%)
Public	9 (45%)	15 (31%)
Private	0 (0%)	3 (6%)
*Rurality* (n, %)		
Metropolitan	10 (50%)	31 (63%)
Regional/rural	10 (50%)	18 (37%)
*Bed number* (mean (SD))	67 (47.6)	85 (57.3)
*Module participation* (n, %)		
Significant organism	12 (60%)	15 (31%)
Staff vaccination	10 (50%)	27 (55%)
Resident vaccination	16 (80%)	24 (49%)
UTI	14 (70%)	17 (35%)

## Abbreviations

IPC: Infection prevention and control
NCAS: National Centre for Antimicrobial Stewardship
NISPAC: National Infection Surveillance Program for Aged Care
RACH: Residential aged care homes
ROSA: Registry of Senior Australians
VICNISS: Victorian Healthcare Associated Infection Surveillance System
